# Leg position effects on the femoral neurovascular bundle location during a direct anterior approach total hip arthroplasty: a radiographic study

**DOI:** 10.1186/s12891-023-06947-0

**Published:** 2023-10-19

**Authors:** Yousuke Tsurumi, Shigeo Hagiwara, Takuro Horikoshi, Hajime Yokota, Ryuna Kurosawa, Koji Matsumoto, Yoshitada Masuda, Yuya Kawarai, Junichi Nakamura, Yawara Eguchi, Sumihisa Orita, Seiji Ohtori

**Affiliations:** 1 Department of Orthopaedics Surgery, Graduate School of Medicine, Chiba University, 1-8-1 Inohana, Chuo-ku, Chiba, 260-8670 Japan; 2 Department of Radiology, Chiba University Hospital, Chiba University Hospital, 1-8-1 Inohana, Chuo-ku, Chiba, 260-8670 Japan; 3 Department of Diagnostic Radiology and Radiation Oncology, Graduate School of Medicine, Chiba University, Chiba University Hospital, 1-8-1 Inohana, Chuo-ku, Chiba, 260-8670 Japan

**Keywords:** Total hip arthroplasty, Direct anterior approach, Neurovascular injury, Magnetic resonance neurography

## Abstract

**Background:**

Femoral neurovascular injury is a serious complication in a direct anterior approach (DAA) total hip arthroplasty. However, dynamic neurovascular bundle location changes during the approach were not examined. Thus, this study aimed to analyze the effects of leg position on the femoral neurovascular bundle location using magnetic resonance imaging (MRI).

**Methods:**

This study scanned 30 healthy volunteers (15 males and 15 females) with 3.0T MRI in a supine and 30-degree hip extension position with the left leg in a neutral rotation position and the right leg in a 45-degree external extension position. The minimum distance from the edge of the anterior acetabulum to the femoral nerve (dFN), artery, and vein were measured on axial T1-weighted images at the hip center level, as well as the angle to the horizontal line of the femoral nerve (aFN), artery (aFA), and vein from the anterior acetabulum.

**Results:**

The dFN in the supine position with external rotation was significantly larger than supine with neutral and extension with external rotation position (20.7, 19.5, and 19.0; p = 0.031 and 0.012, respectively). The aFA in supine with external rotation was significantly larger than in other postures (52.4°, 34.2°, and 36.2°, p < 0.001, respectively). The aFV in supine with external rotation was significantly larger than in supine with a neutral position (52.3° versus 47.7°, p = 0.037). The aFN in supine and external rotation was significantly larger than other postures (54.6, 38.2, and 33.0, p < 0.001, respectively).

**Conclusions:**

This radiographic study revealed that the leg position affected the neurovascular bundle location. These movements can be the risk of direct neurovascular injury or traction.

## Background

Neurovascular injury following total hip arthroplasty (THA) is not a common complication but can be critical [1–3]. A femoral nerve injury can cause weakness of the quadriceps femoris muscle with knee extension deficit, pain, and numbness [2]. Vascular injury can cause life-threatening conditions and amputation [4]. Increasing evidence revealed that anticoagulation, excessive limb lengthening, acetabular rim retractor placement, and dynamic hip motion during exposure lead to neurovascular bundle injury [5]. Therefore, the surgeon should be familiar with the neurovascular bundle location for each approach to prevent injuries.

The direct anterior approach (DAA) for hip arthroplasty has become popular, despite the relatively higher rate of complications, including femoral nerve injury [6]. Several anatomical and radiological studies reported the femoral neurovascular bundle location in the DAA [7–9], but these studies evaluated the static anatomy. The influence of leg position during surgery is not considered in these studies, although extension and external rotation of the hip are required during the exposure of the acetabulum and femur in the DAA. The dynamic change of nerve location during the posterior approach was reported [10], and the sciatic nerve grew closer to the surgical field with hip flexion. There is limited knowledge regarding the change of the neurovascular bundle in the DAA.

We hypothesized that the dynamic leg motion during DAA affects the location of the femoral neurovascular bundle. Therefore, the present study analyzed the femoral artery, vein, and nerve location using a novel method of magnetic resonance (MR) neurography.

## Methods

### Study population

This prospective radiographic study performed in our institute enrolled 30 healthy volunteers (15 males and 15 females). Their demographic data was shown in Table 1. The volunteers had no history of hip pain, trauma, or stiffness and never underwent hip surgery. The study protocol complied with the Helsinki Declaration and was approved by the institutional review board, and all participants gave written informed consent before enrollment.

**Table 1:** Participant Demographics

Variables	Mean	Range
Age at imaging (years)	28.1	(23–41)
Sex (no. [%])		
Men	15	50%
Women	15	50%
Height (cm)	165	(146–179)
Weight (kg)	58	(38–75)
Body mass index (kg/m^2^)	21	(17–25)
Femoral head (mm)	47.4	(43–53)

### MR imaging (MRI)

**Fig. 1:**
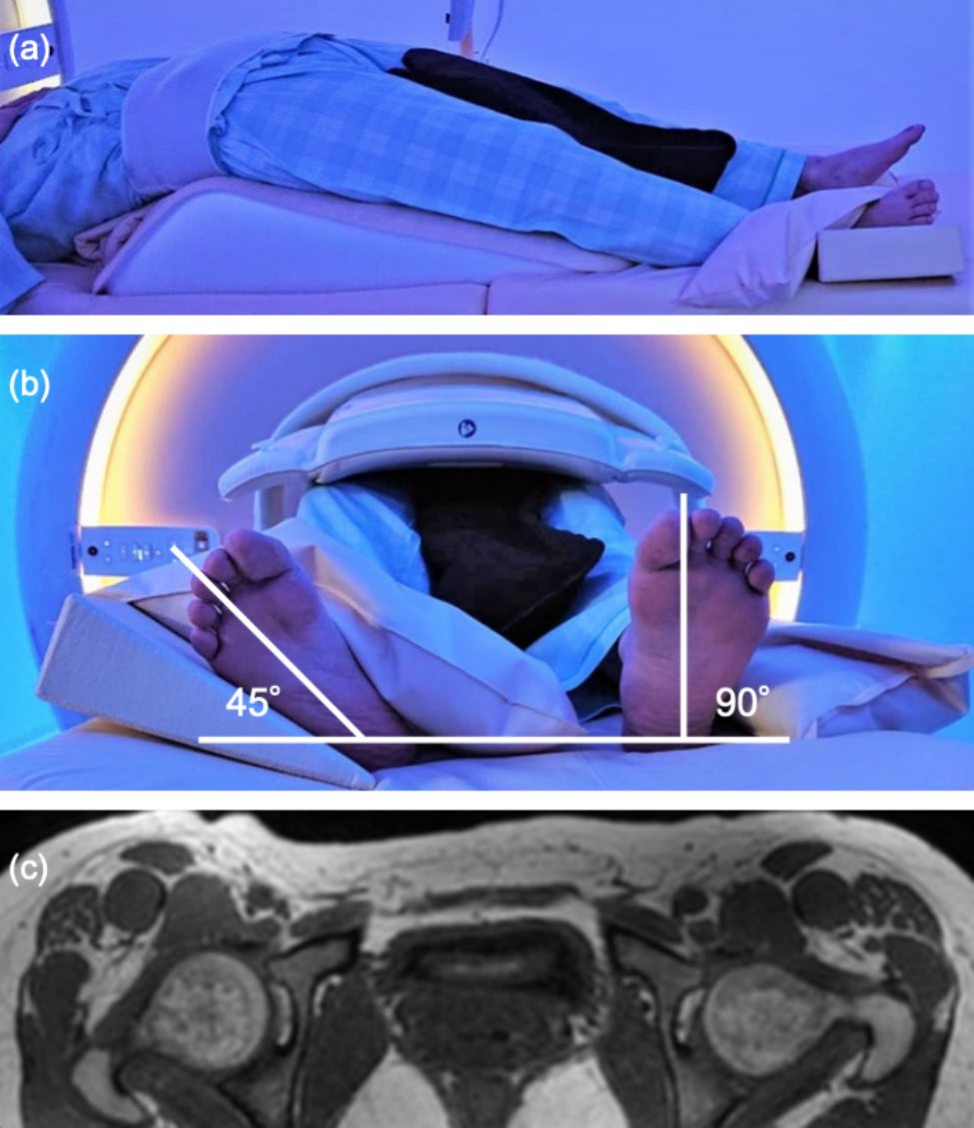
Schematic of the subjects during magnetic resonance (MR) imaging (a) (b). MR image showing the right hip in external rotation and the left hip in neutral position (c)

The bilateral hip joints of each participant were imaged using a 3.0 T MRI system (Ingenia, Philips, Best, Netherlands) with a 16-channel torso coil. Participants were scanned with MRI in the supine position and 30-degree hip extension positions. The matless triangle was used to hold the extension leg position during the examination. The left leg was held in a neutral rotation position and the right whole leg in a 45-degree external extension position (Fig. 1). Examination in the supine position was performed first, followed by the extension position on the same day.

**Fig. 2:**
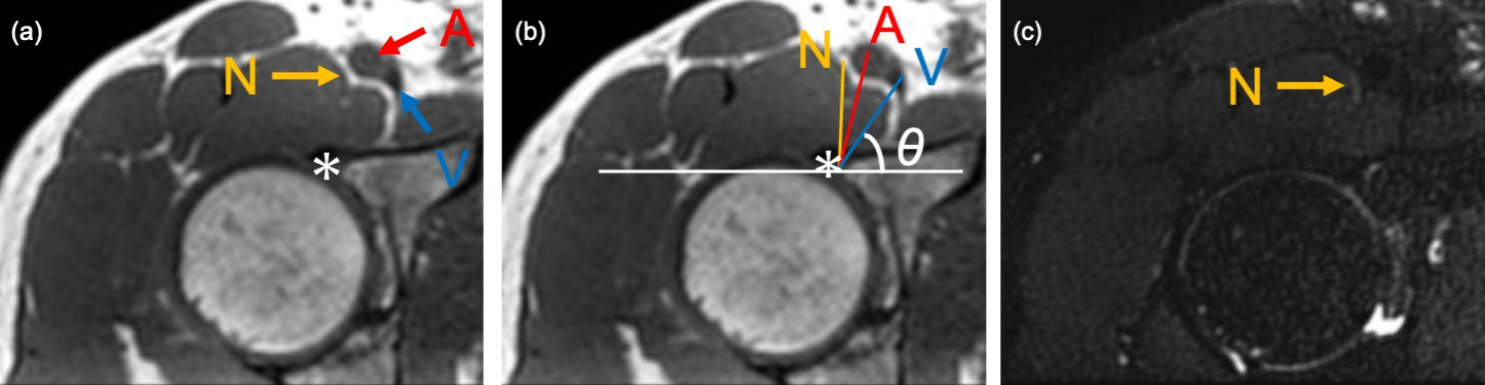
Magnetic resonance (MR) image (a) showing the femoral artery (A), femoral vein (V), and femoral nerve (N) as well as the anterior commissure (*). MR image showing the distance and angle measurements of the femoral neurovascular bundle from the acetabulum (b). Nerve-enhanced image showing the location of (N) (c)

MRI examinations consisted of a coronal three-dimensional (3D) T1-weighted and 3D-Nerve VIEW (Fig. 2) [11] and reconstructed in axial planes to visualize the femoral nerve. The coronal plane was defined along with the anterior pelvic plane to avoid body tilt by the influence of leg position. An advanced MR neurography sequence, 3D-NerveVIEW, consists of a three-dimensional (3D) T2-weighted image, a fat-saturated pulse, and an improved motion-sensitized driven equilibrium (iMSDE) pre-pulse for suppressing blood signals [12, 13]. It can provide neurography with high contrast and resolution. MR parameter were as follows: coronal T1-weighted images (acquisition type = 3D, fat-saturation = mDIXON, repetition time = 4.2 ms, echo time = 1.4/2.4 ms, matrix = 208 × 276, field of view = 300 × 392 mm^2^, slice thickness = 1.4 mm, slice gap = 0.7 mm, voxel resolution = 1.44 × 1.44 × 1.44 mm^3^, slice number = 160); and coronal 3D-NerveVIEW (acquisition type = 3D, fat-saturation = Spectral attenuated inversion recovery(SPAIR), iMSDE duration = 50 ms, repetition time = 2200 ms, echo time = 128 ms, matrix = 288 × 353, field of view = 300 × 370 mm^2^, slice thickness = 1 mm, slice gap 0.5 mm, voxel resolution = 1.0 × 1.0 × 1.0 mm^3^, slice number = 210, acquisition time = 8 min 06 s).

### Distance and direction measurements

The minimum distance and angle of the femoral neurovascular bundle from the acetabulum were measured at the level of the hip center in the axial image. The minimum distance from the edge of the anterior acetabulum to the center point of the femoral nerve (dFN), femoral artery (dFA), and femoral vein (dFV) were measured on axial T1-weighted images using a digital caliper tool Osirix MD (Pixmeo SARL, Geneve, Switzerland) (Fig. 2). Axial 3D-NerveVIEW images were used to identify the femoral nerve. The angle from the edge of the anterior acetabulum to the center point of the femoral nerve (aFN), femoral artery (aFA), and femoral vein (aFV) were also measured on axial T1-weighted images. The transverse line is parallel to the bony structure because the images were obtained according to the anterior pelvic plane.

Measurement was performed in the supine position with the left hip in a neutral rotation position and the right hip in an external rotation position of the hip. Measurement was also performed in the leg extension position with the right hip in an external rotation position. The diameter of the femoral head was also measured as a reference for body size. Each measurement was independently conducted by two board-certified radiologists (TH and HY) and was averaged. Interobserver reliability was tested for each measurement using the intraclass correlation coefficient (ICC).

### Statistical analysis

Data are expressed as case numbers or means with a range. The distance and angle of the femoral neurovascular bundle from the acetabulum were compared between each posture using the repeated measures analysis of variance followed by a Bonferroni correction. The ICC was calculated for the interobserver reliabilities of each parameter. An ICC value of 1 was considered perfect reliability; >0.80 was very good; >0.60 was good; and > 0.40 was moderate [14]. A two-sided *P-*value of < 0.05 was considered statistically significant, and all analyses were performed with SAS Version 9.4 for Windows (SAS Institute Inc., Cary, NC, USA).

## Results

**Fig. 3:**
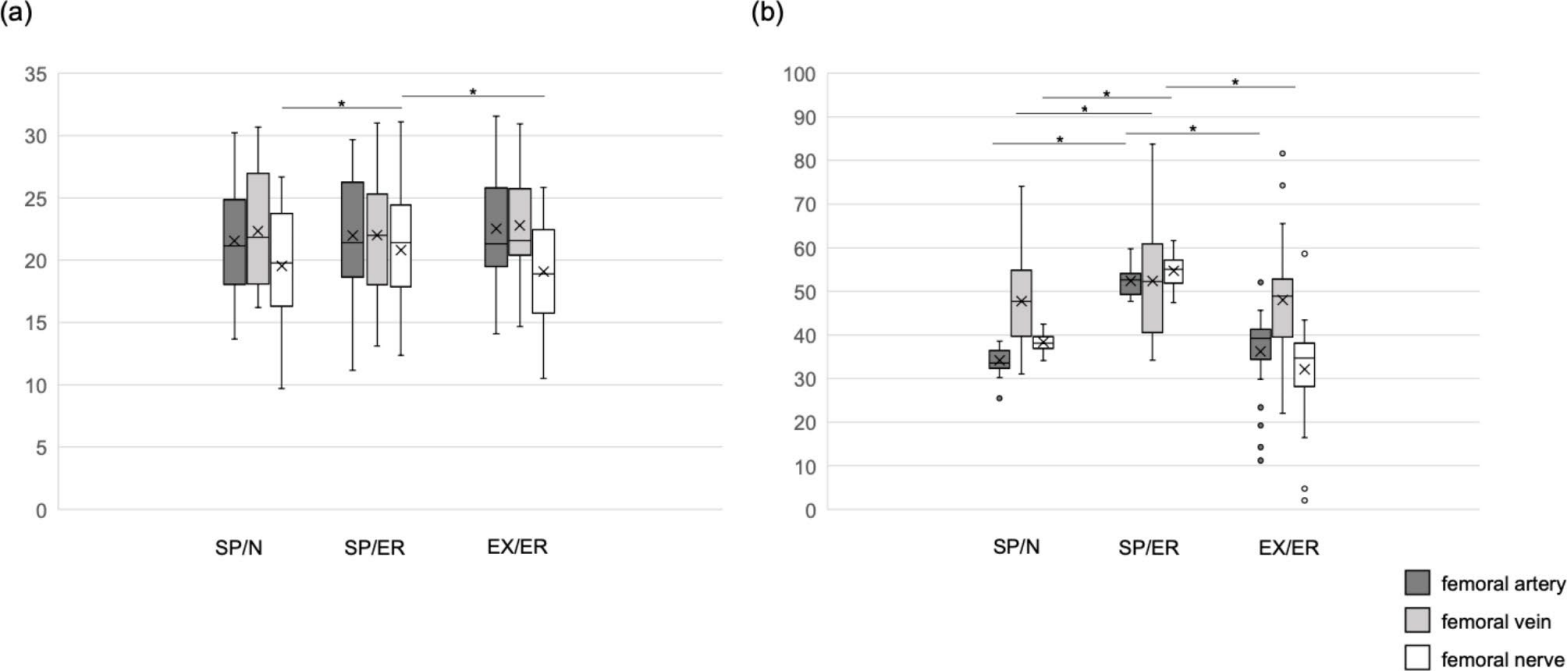
Boxplots display the distance (a) and angle (b) of the neudovascular bundle from the acetabulum. Gray box indicates the femoral artery; light gray box indicates the femoral vein; and white box indicates the femoral nerve. *p < 0.05

Table 2; Fig. 3 show the distance and angle of the femoral neurovascular bundle from the acetabulum. No statistical difference was found in dFA and dFV between each posture. The dFN was significantly larger in the supine with external rotation position than in the supine with neutral and extension with external rotation position (p = 0.031, 0.012, respectively).

**Table 2:** Distances and Angles of the neurovascular bundle from the acetabulum

		SP/N	SP/ER	EX/ER	ICC
Distance (mm)	dFA	21.5 (19.9–23.1)	21.9 (20.2–23.6)	22.5 (20.9–24.1)	0.98 (0.96–0.99)
	dFV	22.3 (20.6–24.0)	22.0 (20.2–23.8)	22.7 (21.1–24.4)	0.93 (0.88–0.96)
	dFN	19.5 (17.9–21.1)	20.7 (18.9–22.6)	19.0 (17.4–20.6)	0.92 (0.88–0.95)
Angle (°)	aFA	34.2 (33.4–35.1)	52.4 (51.2–53.5)	36.2 (32.9–39.5)	0.99 (0.98–0.99)
	aFV	47.7 (43.9–51.6)	52.3 (47.7–57.0)	48.9 (43.6–54.3)	0.97 (0.94–0.98)
	aFN	38.2 (37.3–39.0)	54.6 (53.3–55.9)	33.0 (27.6–38.4)	0.98 (0.97–0.99)

(95% confidence interval)

SP/N: supine/neutral position SP/ER: supine/external rotation EX/ER: extension/external rotation ICC: intraclass correlation coefficients

The aFA in supine with external rotation was significantly larger than in other postures (p < 0.001, respectively). The aFV in supine with external rotation was significantly larger than in supine with a neutral position (p = 0.037). The aFN in supine and external rotation was significantly larger than other postures (p < 0.001, respectively).

The ICCs for interobserver reliabilities of distance and angle measurement are shown in Table 2. All these values indicated perfect reliability.

## Discussion

This study investigated the location of the femoral artery, vein, and nerve using MRI in the dynamic leg motion during DAA. The radiographic assessments were reliable, and the neurovascular bundle location was affected by the leg position. The change of the angle by external rotation and extension was apparent although the change of the distance in the femoral nerve may be clinically small.

The incidence of femoral nerve injury in primary THA via an anterior approach ranges from 0.08 to 1.94% [15, 2, 16], whereas that of vascular complication ranges from 0.09 to 0.4% [4, 17, 7]. Anticoagulation, excessive limb lengthening, and acetabular rim retractor placement are associated with neurovascular injury [18, 2]. A cadaveric study reported the inferior retractor placements to the acetabulum is at risk of neurovascular injury and the safe zone of anterosuperior acetabular retractor placement for DAA [9]; however, the measurement was only in a static position. The retractor is usually placed on the anterior acetabular rim during the exposure; therefore, we defined the edge of the anterior acetabulum as a reference point for radiographic assessment.

The femoral nerve visualization was not easy with conventional MRI because the signal intensity of the nerve was comparable to that of the attached iliopsoas muscle. The femoral nerve is sometimes buried in the muscle and is difficult to identify with regular sequences. 3D-NerveVIEW suppresses the signals of the background structures, including blood vessels, muscles, and fat, resulting in nerve signal enhancement [19]. The interobserver reliabilities were almost perfect in this study, and the measurement was consistent with an anatomical study [20]. Additionally, images were acquired along the anterior pelvic plane and reconstructed to fix the coordinate system of the pelvis. This may contribute to the reliability of measurement.

Femoral blood vessel branches at the inguinal ligament level and runs on the iliopsoas muscle along with the femoral nerve. The nerve, artery, and vein are located from lateral to medial, and the femoral nerve is the closest to the hip joint [21]. An anatomical study reported that the femoral nerve was closest to the acetabular rim at a 90° (3 o’clock) position and the distance from the acetabular rim was positively associated with the iliopsoas muscle thickness [20]. Radiographic evaluation using MRI revealed that the distance between the femoral nerve and the hip joint is associated with a cross-sectional area of the iliopsoas muscle and became smaller in the case of muscle atrophy [8]. Another MRI study reported the difference in neurovascular geometry in the supine and lateral positions, and the distance of neurovascular bundle from the hip joint became closer in supine position [22]. They claimed that the risk of neurovascular injury was higher in the supine position. These studies suggest that the neurovascular bundle location is affected by multiple factors. To our knowledge, no prior study evaluated the effect of the leg position on the femoral neurovascular bundle in the DAA.

The present study revealed a significant change in the neurovascular bundle location in terms of the distance and angle according to the leg position, especially in supine with external rotation. The change in the angle was apparent in the nerve, artery, and vein although the change in the distance was clinically small. The neurovascular bundle moved from medial to lateral in the supine position with external rotation. The iliopsoas muscle attaches to the lesser trochanter and rotates the hip joint externally [21]. This motion may affect the change of neurovascular bundle location. The hip joint rotates externally at approximately 45° during the acetabular exposure; thus, the surgeon should pay attention to preventing the lateral movement of the retractor placement. The artery and nerve moved medially with the external rotation in the extension position. This may be attributed to the traction of these structures. Avoiding long-time exposure of the femur would be better based on the neurovascular protection because of the traction force. The result also can be applicable for anterolateral approach in supine position.

This study has several limitations. First, the participants were young healthy volunteers. THA is performed for elderly patients with osteoarthritis. Muscle atrophy and joint contracture may affect the neurovascular bundle movement. Second, the femoral head was not removed because it was a radiographic study. The femoral head was removed during the hip exposure, which allows complete external rotation, and the soft tissue tension can change. The difference can affect the distance and angle measurements.

## Conclusions

Our study suggests that the external rotation and extension of the hip affects the femoral artery, vein, and nerve locations. These movements can be the risk of direct injury or traction. Therefore, recognizing the movement of the neurovascular bundle during hip exposure in supine position may be important for protection.

## Data Availability

This work was performed at Chiba University Hospital. The datasets generated and analysed during the current study are not publicly available due to limitations of ethical approval involving the patient data and anonymity but are available from the corresponding author on reasonable request.

## List of abbreviations

THA: total hip arthroplasty
DAA: direct anterior approach
MRI: magnetic resonance imaging
FA: femoral artery
FV: femoral vein
FN: femoral nerve
ICC: intraclass correlation coefficient

## References

[1] Ratliff AH (1985). Arterial injuries after total hip replacement. J Bone Joint Surg Br.

[2] Fleischman AN, Rothman RH, Parvizi J (2018). Femoral nerve palsy following total hip arthroplasty: incidence and course of recovery. J Arthroplasty.

[3] An S, Shen H, Feng M, Li Z, Wang Y, Cao G (2018). Femoral artery injury during total hip arthroplasty. Arthroplast Today.

[4] Alshameeri Z, Bajekal R, Varty K, Khanduja V (2015). Iatrogenic vascular injuries during arthroplasty of the hip. Bone Joint J.

[5] Vajapey SP, Morris J, Lynch D, Spitzer A, Li M, Glassman AH (2020). Nerve injuries with the Direct Anterior Approach to total hip arthroplasty. JBJS Rev.

[6] Woolson ST (2020). A survey of Hip Society surgeons concerning the direct anterior approach total hip arthroplasty. Bone Joint J.

[7] Davis ET, Gallie PA, James SL, Waddell JP, Schemitsch EH (2010). Proximity of the femoral neurovascular bundle during hip resurfacing. J Arthroplasty.

[8] Yoshino K, Hagiwara S, Nakamura J, Horikoshi T, Yokota H, Shimokawa K (2021). The distance between the femoral nerve and anterior acetabulum is significantly shorter in hip osteoarthritis than in non-osteoarthritis hip. BMC Musculoskelet Disord.

[9] Sullivan CW, Banerjee S, Desai K, Smith M, Roberts JT (2019). Safe zones for anterior acetabular retractor placement in direct anterior total hip arthroplasty: a cadaveric study. J Am Acad Orthop Surg.

[10] Kanawati AJ, Narulla RS, Lorentzos P, Facchetti G, Smith A, Stewart F (2015). The change in position of the sciatic nerve during the posterior approach to the hip. Bone Joint J.

[11] Eilander W, Harris SJ, Henkus HE, Cobb JP, Hogervorst T (2013). Functional acetabular component position with supine total hip replacement. Bone Joint J.

[12] Yoneyama M, Takahara T, Kwee TC, Nakamura M, Tabuchi T (2013). Rapid high resolution MR neurography with a diffusion-weighted pre-pulse. Magn Reson Med Sci.

[13] Kasper JM, Wadhwa V, Scott KM, Rozen S, Xi Y, Chhabra A (2015). SHINKEI–a novel 3D isotropic MR neurography technique: technical advantages over 3DIRTSE-based imaging. Eur Radiol.

[14] Walter SD, Eliasziw M, Donner A (1998). Sample size and optimal designs for reliability studies. Stat Med.

[15] Farrell CM, Springer BD, Haidukewych GJ, Morrey BF (2005). Motor nerve palsy following primary total hip arthroplasty. J Bone Joint Surg Am.

[16] Schmalzried TP, Noordin S, Amstutz HC (1997). Update on nerve palsy associated with total hip replacement. Clin Orthop Relat Res.

[17] Post ZD, Orozco F, Diaz-Ledezma C, Hozack WJ, Ong A (2014). Direct anterior approach for total hip arthroplasty: indications, technique, and results. J Am Acad Orthop Surg.

[18] Fox AJ, Bedi A, Wanivenhaus F, Sculco TP, Fox JS (2012). Femoral neuropathy following total hip arthroplasty: review and management guidelines. Acta Orthop Belg.

[19] Hiwatashi A, Togao O, Yamashita K, Kikuchi K, Kamei R, Momosaka D (2017). Lumbar plexus in patients with chronic inflammatory demyelinating polyneuropathy: evaluation with 3D nerve-sheath signal increased with inked rest-tissue rapid acquisition of relaxation enhancement imaging (3D SHINKEI). Eur J Radiol.

[20] Yoshino K, Nakamura J, Hagiwara S, Suzuki T, Kawasaki Y, Ohtori S (2020). Anatomical implications regarding femoral nerve palsy during a direct anterior approach to total hip arthroplasty: a cadaveric study. J Bone Joint Surg Am.

[21] Netter FH. Atlas of human anatomy. 7th ed. ed: Elsevier; 2019.

[22] Takada R, Jinno T, Miyatake K, Hirao M, Yoshii T, Kawabata S (2021). Does surgical body position influence the risk for neurovascular injury in total hip arthroplasty? A magnetic resonance imaging study. Orthop Traumatol Surg Res.

